# Letter to the Editors: Comparing surgical interventions for intertrochanteric hip fracture by blood loss and operation time: a network meta-analysis

**DOI:** 10.1186/s13018-019-1195-9

**Published:** 2019-05-22

**Authors:** Peng Luo, Ding Xu, Wei Jun Guo

**Affiliations:** 1 Department of Orthopedics, The Second Affiliated Hospital and Yuying Children’s Hospital of Wenzhou Medical University, Wenzhou, China; 2 Department of Orthopedics, Shangyu People’s Hospital of Shaoxing City, Shaoxing, China

Recently, we read the publication written by Zhengan Hao and his colleagues with great interest (Hao Z, Wang X and Zhang X. Comparing surgical interventions for intertrochanteric hip fracture by blood loss and operation time: a network meta-analysis. J Orthop Surg Res. 2018 Jun 22;13(1):157). The authors carried out a meta-analysis to help surgeons choose the appropriate treatments for patients with intertrochanteric hip fractures through the perspectives of blood loss and operation times. However, we have to point out several deficiencies of this study, as some of them might lead to the bias of conclusions. Firstly, we noted that the numerical sequences of included studies in the text were inconsistent with that in the reference list. It would make readers hard to find the reference they are interested in. Secondly, in our opinion, it might be better to exclude the publication written by Garg et al. (2011) [1] in this meta-analysis, as it was retracted in 2012 due to infringements of professional ethical codes [2]. Thirdly, it is worth to note that there might be an overlapping part of patients in this meta-analysis, because three articles involved in this study actually come from the same department during an overlapping period [3–5]. Finally, the authors stated that they extracted the blood loss from each study by measuring the change from baseline. In our view, the authors should describe more details on this issue, because the measurement methods of blood loss among the studies were inconsistent. For example, Kosygan et al*.* used the postoperative fall of Hb (g%) to present the blood loss [6]. In Ahrengart et al*.*’s study, the blood loss was composed by different parts, including hemoglobin at admission, perioperative blood loss, blood loss to drainage, number of blood transfusions, and hemoglobin 1 week postoperatively [7]. However, Changyou et al. only reported the intraoperative bleeding in their study [8]. As the deficiencies we mentioned above, the conclusions of this review might be limited.
